# Interrupting long periods of sitting: good STUFF

**DOI:** 10.1186/1479-5868-10-1

**Published:** 2013-01-02

**Authors:** Geert M Rutten, Hans H Savelberg, Stuart JH Biddle, Stef PJ Kremers

**Affiliations:** 1 Department of Health Promotion, NUTRIM, Maastricht University, P.O. Box 616, 6200 MD, Maastricht, The Netherlands; 2 Department of Human Movement Sciences, NUTRIM, Maastricht University, P.O. Box 616, 6200 MD, Maastricht, The Netherlands; 3 Loughborough University, School of Sport, Exercise and Health Sciences, Ashby Road, Loughborough Leicestershire, United Kingdom

**Keywords:** Sedentary behavior, Physical activity, Sitting time reduction, Health promotion, Dissemination

## Abstract

There is increasing evidence that sedentary behaviour is in itself a health risk, regardless of the daily amount of moderate to vigorous physical activity. Therefore, sedentary behaviour should be targeted as important health behaviour.

It is known that even relatively small changes of health behaviour often require serious efforts from an individual and from people in their environment to become part of their lifestyle. Therefore, interventions to promote healthy behaviours should ideally be simple, easy to perform and easily available. Since sitting is likely to be highly habitual, confrontation with an intervention should almost automatically elicit a reaction of getting up, and thus break up and reduce sitting time. One important prerequisite for successful dissemination of such an intervention could be the use of a recognisable term relating to sedentary behaviour, which should have the characteristics of an effective brand name. To become wide spread, this term may need to meet three criteria: the “Law of the few”, the “Stickiness factor”, and the “Power of context”. For that purpose we introduce STUFF: Stand Up For Fitness. STUFF can be defined as “interrupting long sitting periods by short breaks”, for instance, interrupting sitting every 30 min by standing for at least five minutes.

Even though we still need evidence to test the health-enhancing effects of interrupted sitting, we hope that the introduction of STUFF will facilitate the testing of the social, psychological and health effects of interventions to reduce sitting time.

## Background

Diseases originating from lack of physical activity are a growing problem in the world. So far interventions to cope with this have focused on stimulating people to engage in daily moderate-to-vigorous physical activity (MVPA) [1]. However, an increasing number of studies have recently provided evidence that sedentary behaviour is in itself a health risk, regardless of the daily amount of MVPA [2,3]. This implies that excessive sitting cannot be wholly compensated for by half an hour of MVPA, and several scholars have suggested a shift in scientific focus to include the physiology of “inactivity” (sedentary) as well as exercise [2,4,5]. If we slice up a day into periods of MVPA, light PA, sedentary behaviour and sleep, we see that a large proportion of the time is taken up by sedentary pursuits, such as TV viewing, car driving and computer use. Moreover, sedentary time is more likely to be replaced by light PA rather than MVPA. It thus makes sense to target sedentary behaviour as an important health behaviour.

## What to do about it

Next to increasing MVPA, interventions to promote a healthy lifestyle simultaneously need to aim at limiting the time spent sitting [6]. The rationale behind this advice is that longer episodes of sedentary behaviour evoke catabolic processes [7-9]. Although little is known about the thresholds for time spent sitting or how long sitting should be interrupted before it evokes health consequences, evidence shows that short breaks in sitting may prevent these degrading processes [2,10,11]. Given the physiological reactions of the body that are induced just by standing up, individuals with lifestyle-related diseases may gain much by regularly interrupting or reducing their sitting time [12].

## How to get there

A well-known problem is that the availability of behavioural alternatives with positive impact on health does not automatically result in uptake of these behaviours by the target population [13,14]. Even relatively small changes in behaviour often require a serious effort from an individual and from people in their environment to become part of their lifestyle. Nevertheless, newly acquired health behaviours that have become part of habitual daily routines are most likely to be sustained over time and to result in positive health consequences. Hence, it would be ideal if an intervention managed to introduce habits promoting the interruption of prolonged sitting which become part of daily routines. Such an intervention should ideally be simple, should not require much cognitive energy, and should be easy to perform. Moreover, it should be widespread, easily available and recognisable. Being confronted with it should almost automatically elicit a reaction of getting up, and thus break up and reduce sitting time. In this respect, sedentary behaviour may be different from physical activity. Sitting is likely to be highly habitual, with little or no conscious processing, whereas MVPA requires higher levels of conscious processing and planning.

## Spreading the word

In addition to optimal intervention content, one important prerequisite for successful dissemination could be the use of a recognisable term relating to sedentary behaviour. This should have the characteristics of an effective brand name that users pass on to others and are willing to accept and “own” as part of their individual identities. Such a “brand” should have true and meaningful value and should be associated with positive consequences, which may be reflected in it being turned into a commonly used verb. Although some examples are available from the commercial world, such as Google or Twitter, examples from the social or health world are less common. If the term is to become widespread, it may need to meet three criteria [15]. The first is the “Law of the few”, which means that it requires people with a particular set of social gifts to spread it (connectors, experts and salespeople). Second, it should have the “Stickiness factor”, implying that the term has to be memorable and drive people to action. Finally, there is the “Power of context”, which indicates that its successful dissemination depends on conditions and circumstances of time and place.

Hence, health promoters should use their networks and reach out to their connectors, experts and salespeople to spread the term, and have it transformed into a verb that is generally recognised and used. It requires that the term sticks in people’s memories and induces them to get up from their chairs. In view of the context, which in this case includes evidence for the relation between physical inactivity and various non-communicable diseases [12], and the barriers that prevent, for example, obese individuals from engaging in MVPA [16], we feel there are sufficient reasons to launch the idea at this point in time. Time will tell if we are right.

## Good STUFF

Explicitly labelling or “branding” a behavioural goal or performance objective [17] will assist the dissemination of a health promotion message. We would therefore like to introduce the acronym STUFF: STand Up For Fitness. The term “fitness” is used to express the general social, psychological and health promoting effect of less sitting. STUFF can be defined as “interrupting long sitting periods by short breaks”, for instance, interrupting sitting every 30 min by standing for at least five minutes.

The ideal would be to have the name STUFF become part of the public consciousness. In two years from now, we would hope to hear people say: “OK, the meeting has now been going on for half an hour, time to stuff”, or “Children, this test will take one hour, including stuff”; or “Ladies and gentlemen, we’re going to have a commercial break, take your stuff”. People could even say “Let’s stuff for a moment”, or have an additional reason to mimic colleagues that get up to applaud presenters in conferences with “Hey, that’s great stuff you got there!” It could even be translated into a cover of the hit song by Brian Ferry: “let‘s stuff together, c’mon, c’mon, let’s stuff together” or in Christmas festivities: “[…], o what fun it is to stuff in a one-horse open sleigh”. And ideally, instead of people only raising their eyebrows, it would make them raise their body from the chair.

We have gained some preliminary experience with the implementation of STUFF during lectures, using a triggering image on the lecture slides that pops up every 30 min. The lecture continues during such a STUFF, while students are standing for 5 min. This has evoked positive reactions from students, who reported “It was fun, a change from what we’re used to”, “It actually helped me maintain my concentration”, and “I had to get used to it, because my paper and pencil were still on my desk the first time, but at the next STUFF I just took my notes while standing.” Admittedly, these were health science students and our initial experiences are thus not generalisable to the larger population, but it may indicate that STUFF is good stuff for health promoters. That said, these are just preliminary ideas and we still need evidence to test the health-enhancing effects of interrupted sitting. We hope that the introduction of STUFF will facilitate the testing of social, psychological and health effects of interventions to reduce sitting time.

## Competing interests

The authors declare that they have no competing interests.

## Authors’ contributions

GMR and SPJK conceived the idea. GMR wrote the paper and was responsible for adjustments on the basis of comments of the co-authors. HHS, SJHB and SPJK contributed equally to the paper by reviewing it and proposing adjustments for improvement. All authors read and approved the final manuscript.
